# Reliability assessment of methylthioadenosine phosphorylase immunohistochemistry as a surrogate biomarker for *CDKN2A* homozygous deletion in adult-type IDH-mutant diffuse gliomas

**DOI:** 10.1093/jnen/nlad109

**Published:** 2023-12-18

**Authors:** Fatma Gundogdu, Berrin Babaoglu, Figen Soylemezoglu

**Affiliations:** Department of Pathology, Hacettepe University, Ankara, Turkey; Department of Pathology, Hacettepe University, Ankara, Turkey; Department of Pathology, Hacettepe University, Ankara, Turkey

**Keywords:** Cyclin-dependent kinase 2A, IDH-mutant diffuse glioma, Interobserver agreement, Methylthioadenosine phosphorylase (MTAP), Surrogate biomarker

## Abstract

According to the 2021 World Health Organization classification of brain tumors, astrocytomas containing a *CDKN2A/B* homozygous deletion (HD) are designated as grade 4 even when no microvascular proliferation and/or necrosis is present. In this study, we aimed to investigate the relationship between *CDKN2A* HD and loss of methylthioadenosine phosphorylase (MTAP) expression in adult-type IDH-mutant gliomas and to assess the sensitivity and specificity of MTAP immunohistochemistry (IHC) along with interobserver agreement as a surrogate biomarker for *CDKN2A* HD. Eighty-eight astrocytomas and 71 oligodendrogliomas cases that were diagnosed between 2014 and 2021 at Hacettepe University were selected and tissue microarrays were conducted to perform *CDKN2A* fluorescence in situ hybridization and MTAP IHC. Twenty-five (15.7%) cases harbored *CDKN2A* HD. MTAP loss was detected in 28 (15.7%) cases by the first observer and 27 (17%) cases by the second observer. The sensitivity and specificity of MTAP were calculated as 88% and 95.52%-96.27% for 2 observers. A very good/perfect agreement was noted between the observers (Cohen kappa coefficient = 0.938). Intratumoral heterogeneity was observed in 4 cases. MTAP IHC was found to be a reliable surrogate biomarker as a possible alternative to *CDKN2A* HD identification with a high sensitivity and specificity along with high interobserver agreement.

## INTRODUCTION

Diffuse gliomas comprise a significant proportion of primary malignant intracranial tumors. In the 2021 World Health Organization (WHO) classification of central nervous system (CNS) tumors, diffuse glial tumors are categorized under adult and pediatric types (1). In the process that started with the discovery of isocitrate dehydrogenase (*IDH*) *1/2* (*IDH1/2*) mutations in 2009 (2), adult-type diffuse gliomas are divided into different subtypes based on *IDH1/2* mutation and 1p/19q codeletion (3) status. According to the 2021 WHO, they are classified into 3 groups; IDH-mutant astrocytoma, IDH-mutant and 1p/19q co-deleted oligodendroglioma, and IDH-wild type glioblastoma.

Recently, homozygous deletion (HD) of cyclin-dependent kinase inhibitors 2A and 2B (*CDKN2A/B*) genes has emerged as a biomarker that can be used to predict poor prognosis in diffuse astrocytic tumors. Many studies have revealed that *CDKN2A/B* HD is associated with adverse prognosis and reduced survival in IDH-mutant diffuse astrocytic gliomas (4–6). In the “cIMPACT-NOW” published in 2020, it was recommended that astrocytomas containing *CDKN2A/B* HD be considered WHO grade IV (2016) astrocytomas even if there is no microvascular proliferation (MVP) or necrosis (7). Based on these findings, according to the change in the grading of IDH-mutant astrocytomas in the 2021 WHO CNS tumors classification, tumors containing *CDKN2A/CDKN2B* HD are classified as grade 4 even if they do not contain microvascular proliferation and/or necrosis (1). While *CDKN2A/B* HD can be seen in some (<10%) of WHO CNS grade 3 oligodendrogliomas, it is not expected in grade 2 oligodendrogliomas, and its presence has been related to short survival times independent of MVP and necrosis (8). Therefore, *CDKN2A/B* HD can be used as a molecular aid in oligodendroglial tumors that are difficult to grade.

*CDKN2A* and *CDKN2B* genes are adjacent to each other on chromosome 9p21. The *CDKN2A* gene plays a role in encoding p16 and p14 tumor suppressor proteins, while the *CDKN2B* gene encodes p15 protein. In this way, they inhibit the transition of cells from the G1 to the S phase and MDM2 activity that causes p53 degradation (9, 10). *CDKN2A/B* pathway alterations contribute to the development of various malignant tumors. Currently, *CDKN2A* HD can usually be evaluated using molecular methods such as fluorescence in situ hybridization (FISH), comparative genomic hybridization, multiplex ligation-dependent probe amplification, next-generation sequencing, or DNA methylation-based profiling. However, because of the high cost, long reporting period, and difficulty in accessing these sophisticated molecular methods, more accessible and low-cost methods such as immunohistochemical stains are needed.

There are controversial results on the reliability of p16, the loss of which has long been thought to be a possible surrogate marker for *CDKN2A* in glial tumors (11–13). Recently, there have been studies conducted with mesothelioma, various epithelial tumors, meningioma, and pleomorphic xanthoastrocytoma (PXA) in order to use methylthioadenosine phosphorylase (MTAP) immunohistochemical expression loss as a surrogate biomarker to detect *CDKN2A* HD status (14–21). The *MTAP* gene, which is located on chromosome 9p.21 and 165 kb telomeric to the *CDKN2A* gene, shows HD along with *CDKN2A* HD in 80%-90% of cases. The MTAP protein is present in every cell and has a crucial role in adenosine monophosphate and methionine synthesis. When the *MTAP* gene is HD, normally expected cytoplasmic MTAP protein expression is lost and this can be detected by IHC (22, 23). The anticipated role of MTAP IHC in diffuse gliomas, as an alternative to *CDKN2A* HD, has not yet been fully explored; there are only 2 published studies in the literature. In 2020, Satomi et al reported 88% sensitivity and 98% specificity for MTAP IHC in predicting *CDKN2A* HD in IDH-mutant astrocytomas. They also found that loss of MTAP expression was associated with adverse prognosis and shorter survival times similar to *CDKN2A* HD (24). Three years later, Maragkou et al (25) reported that cytoplasmic MTAP expression loss had 100% sensitivity and 97% specificity in their glioma cohort.

With the 2021 WHO classification of brain tumors, the frequent use of expensive methods has become inevitable but since it will not be possible to examine the genomic and proteomic features of all brain tumors, especially in low- and middle-income countries, an approach in which immune surrogate biomarkers can be used would be beneficial.

Our aim in this study was to assess the relationship between *CDKN2A* HD and loss of MTAP expression in adult-type IDH-mutant diffuse gliomas and determine the sensitivity and specificity of MTAP immunohistochemistry (IHC) as a surrogate biomarker for FISH assay detection of *CDKN2A* deletion of 2 independent observers. To the best of our knowledge, this would be the first study to evaluate interobserver agreement and intratumoral heterogeneity of MTAP staining in gliomas, along with its sensitivity and specificity.

## MATERIALS AND METHODS

### Patient selection

This retrospective and observational study had 159 adult-type IDH-mutant diffuse glioma cases that were diagnosed between 2014 and 2021 at Hacettepe University, Department of Pathology (Ankara, Turkiye), and had representative formalin-fixed, paraffin-embedded blocks with an adequate amount of tumor tissue. All hematoxylin and eosin (H&E) and immunohistochemically stained slides were reevaluated by 2 pathologists (F.S. and F.G.), one experienced in neuropathology (F.S.). IDH and 1p/19q status were known in advance for all of the tumors but the *CDKN2A* status was unknown. Tumors were graded according to WHO CNS 2016 at the time of diagnosis without information on *CDKN2A* status. The 159 tumors were divided into 2 groups, that is astrocytoma, IDH-mutant, and oligodendrogliomas, IDH-mutant and 1p/19q co-deleted. The astrocytoma group consisted of 88 tumors belonging to 80 individual patients. Eighty-five tumors had IDH1 (R132H) mutation detected by IHC and 3 immunonegative cases were sequenced and non-R132H mutations in codon 132 of *IDH1* were found in all. The oligodendroglioma group consisted of 71 tumors belonging to 69 individual patients. All of them had 1p/19q codeletion with FISH; 68 had immunohistochemical IDH1 (R132H) positivity; and 3 had *IDH2* mutations that were detected by sequencing. The epidemiological and clinical features including age, gender, and tumor location were recorded.

### Tissue microarray

To prepare tissue microarrays (TMAs), representative tumor paraffin blocks belonging to each case were selected. Two 3-mm-diameter cores of tumor tissue were taken out from the selected blocks with the help of a manual punch arrayer and the cores were placed in new receiver blocks. Eleven TMA blocks were constructed, and 4-μm multiple sections were taken from each. One of them was stained H&E to check the presence of tumoral tissue under a light microscope.

### Immunohistochemistry

**Figure 1.**
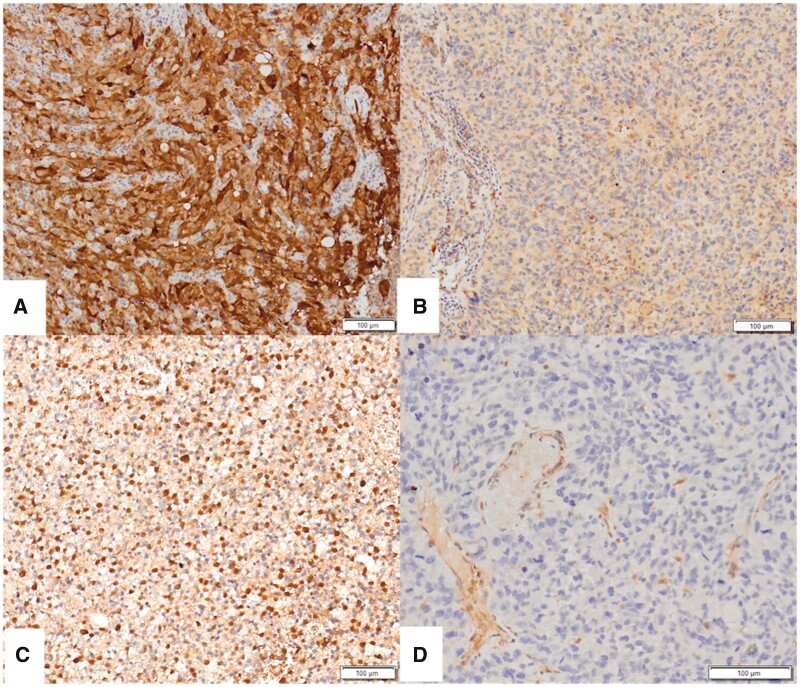
Evaluation of MTAP staining. **(A)** Strong cytoplasmic expression. **(B)** weak cytoplasmic expression. **(C)** Nuclear staining along with cytoplasmic staining. **(D)** Cytoplasmic expression loss with positive internal control staining. All are at 200× magnification.

TMA sections were stained with MTAP antibody (Abcam, Cambridge, United Kingdom, clone EPR6893, Targeted Retrieval Solution, pH 9.0, 1:1000 dilution, 25 minutes incubation) using Leica Bond-Max (Leica Microsystems, Wetzlar, Germany) automated IHC platform, according to the manufacturer’s instructions. As an external control, colon tissues were used in every block. Cytoplasmic staining with or without nuclear staining was accepted as preserved MTAP expression (Fig. 1A–C). Complete loss of cytoplasmic staining with adequate positive internal controls (endothelial, inflammatory, and non-neoplastic glial cells) was accepted as loss of MTAP expression (Fig. 1D). MTAP staining was evaluated and scored by 2 pathologists (F.S. and F.G.) independently for both tumor cores of each case.

### Fluorescence in situ hybridization

For *CDKN2A* FISH analysis, Zytolight SPEC *CDKN2A/CEN 9* Dual Color Probe was used on TMA sections according to the manufacturer’s instructions. CDKN2A FISH analysis was performed on both tumor cores of each case. The prepared slides were examined under a fluorescence microscope. The *CDKN2A* probe (test probe) was expected to give green, and the *CEN9* probe (reference probe) would give orange signal. Two orange and 2 green signals were expected in a normal interphase nucleus. One hundred non-overlapping cells were counted in a dark field. *CDKN2A* HD was defined as the case that the number of cells containing at least one orange signal and missing both green signals was greater than 10%. *CDKN2A* heterozygous deletion was defined as a test probe/reference probe ratio ≤0.8 and the percentage of cells with a reference probe/test probe ratio of 2 was ≥35%. *CDKN2A* amplification was defined as a test probe/reference probe ratio ≥2.

### Statistical analysis

Statistical analyses were performed in SPSS version 25.0 (IBM, Armonk, NY), and p < 0.05 was considered statistically significant. The normal distribution of continuous data was evaluated by visualization of histograms and the Shapiro-Wilk test. Normally distributed continuous data were reported as mean, standard deviation, and categorical data as numbers and percentages. The difference between 2 independent groups of continuous data was evaluated with the independent t-test. Chi-square or Fisher exact test investigated the relationship between categorical variables. Sensitivity, specificity, positive and negative odds ratio, positive and negative predictive ratio, and precision were evaluated with 95% confidence intervals to assess the diagnostic accuracy of the tests. The agreement between the 2 observers was reviewed by the Kappa coefficient and interpreted as follows: <0.2, slight; 0.2 to <0.4, fair; 0.4 to <0.6, moderate; 0.6 to <0.8, substantial/good; 0.8 to <1.0, very good/perfect.

## RESULTS

### Clinical and histopathological features

Clinicopathological findings are summarized in Table 1. In this study, 159 adult-type IDH-mutant diffuse glioma cases belonging to 147 patients were evaluated. Fifty-two (35.3%) of 147 patients were female, and 95 (64.6%) were male. The mean age for astrocytoma was 44.3 and 34.9 years for oligodendroglioma.

**Table 1.** Clinicopathologic parameters

	Astrocytoma (n)	Oligodendroglioma (n)
Sex (n)		
Male	59 (88)	48 (71)
Female	29 (88)	23 (71)
Age (years)		
Mean	34.9	44.3
Location (n)		
Frontal lobe	42 (88)	49 (71)
Temporal lobe	34 (88)	17 (71)
Parietal lobe	11 (88)	5 (71)
Occipital lobe	1 (88)	0 (71)
WHO CNS grade (2016) (n)		
Grade II	35 (88)	28 (71)
Grade III	26 (88)	43 (71)
Grade IV	27 (88)	—
WHO CNS grade (2021) (n)		
Grade 2	33 (88)	28 (71)
Grade 3	18 (88)	43 (71)
Grade 4	37 (88)	—

Seventy-one (44.7%) cases were diagnosed as an oligodendroglioma, IDH-mutant, and 1p/19q codeleted, and 88 (55.3%) as astrocytoma, IDH-mutant. Among cases of astrocytoma, 35 (39.7%) were graded as grade II, 26 (29.5%) as grade III, and 27 (30.6%) as grade IV (IDH-mutant glioblastoma) at the time of diagnosis according to WHO CNS 2016. After the evaluation of *CDKN2A* status, the WHO CNS grade of 10 cases (2 grade II cases, 8 grade III cases) that harbored *CDKN2A* HD were changed to WHO CNS grade 4 IDH-mutant astrocytoma in accordance with WHO CNS 2021. In oligodendroglial tumors, 28 (39.4%) cases were WHO CNS grade 2, and 43 (60.5%) cases were WHO CNS grade 3.

### FISH and IHC results

The results of *CDKN2A* FISH and MTAP IHC are summarized in Table 2. The *CDKN2A* status of 159 tumors was evaluated by FISH. *CDKN2A* HD was detected in 25 (15.7%) of 159 tumors. In astrocytomas, 20 (22.7%) of 88 cases had *CDKN2A* HD, while 5 (7%) of 71 oligodendrogliomas showed this deletion. The 2016 WHO CNS grades of the astrocytomas that harbor *CDKN2A* HD were grade II in 2 tumors, grade III in 8 tumors, and grade IV in 10 tumors. After evaluating *CDKN2A* HD status, the WHO CNS grade of 10 tumors was changed to WHO CNS grade 4 according to the WHO CNS 2021 criteria. In the oligodendrogliomas, none of the WHO CNS grade 2 tumors had *CDKN2A* HD, while 5 (11.6%) of 43 grade 3 cases showed *CDKN2A* HD.

**Table 2.** *CDKN2A* FISH And MTAP IHC results

	All cases (n)	Astrocytoma (n)	Oligodendroglioma (n)
*CDKN2A* (n)			
HD	25 (159)	20 (88)	5 (71)
Normal	134 (159)	68 (88)	66 (71)
1. observer (F.S.) (n)			
MTAP loss	28 (159)	23 (88)	7 (71)
MTAP preserved	131 (159)	65 (88)	64 (71)
2. observer (F.G.) (n)			
MTAP loss	27 (159)	22 (88)	7 (71)
MTAP preserved	132 (159)	66 (88)	64 (71)

Regarding MTAP expression, 159 tumors were scored by 2 observers. The first observer (F.S.) assessed 28 (15.7%) of them, and the second observer (F.G.) assessed 27 (17%) of them as having a loss of MTAP expression. In the astrocytoma group, the first observer detected MTAP loss in 23 (23.9%) of the tumors, and the second observer detected MTAP loss in 22 (23.9%) of them. In the oligodendroglioma group, MTAP loss was detected in 7 (9.9%) of 73 tumors evaluated. There were only 3 cases that did not show interobserver agreement due to heterogenous staining patterns characterized by the intermingling of stained and non-stained cells and in which identifying which cells were neoplastic and which were not was not entirely clear on first evaluation. However, after the re-evaluation of these MTAP stainings with their H&E counterparts, it was decided that this heterogenous staining reflected intratumoral heterogeneity.

*CDKN2A* heterozygous deletion was found in 3 of 88 astrocytomas (3.4%) while 3 cases (3.4%) showed *CDKN2A* amplification. In none of these cases, MTAP expression loss was observed. No *CDKN2A* heterozygous deletion or amplification was detected in oligodendrogliomas.

When the interobserver agreement for the detection of MTAP loss was evaluated with Cohen kappa coefficient, a very good/perfect agreement of 0.938 was found between the 2 observers.

### Correlation between *CDKN2A* HD and loss of MTAP expression

The comparison of *CDKN2A* HD and MTAP expression is shown in Table 3. When the relationship between the presence of *CDKN2A* HD and loss of MTAP expression in both observers was evaluated with the Fisher exact test, it was found that MTAP loss was higher in the presence of *CDKN2A* HD (p < 0.001).

**Table 3.** Comparison of *CDKN2A* HD and MTAP expression

	All tumors (%)		Astrocytomas (%)		Oligodendrogliomas (%)	
	*CKDN2A* HD	*CKDN2A* normal	*CKDN2A* HD	*CKDN2A* normal	*CKDN2A* HD	*CDKN2A* normal
Observer (F.S.)						
MTAP lost (n)	22 (88%)	6 (4.5%)	18 (90%)	3 (4.4%)	4 (80%)	3 (4.5%)
MTAP preserved (n)	3 (12%)	128 (95.5%)	2 (10%)	65 (95.6%)	1 (20%)	63 (95.4)
Sensitivity	88%		90%		80%	
Specificity	95.52%		95.59%		95.45%	
Positive predictive value	78.54%		85.71%		57.14%	
Negative predictive value	97.71%		97.01%		98.44%	
Observer (F.G.)						
MTAP lost (n)	22 (88%)	5 (3.7%)	17 (85%)	3 (4.4%)	5 (100%)	2 (3.1%)
MTAP preserved (n)	3 (12%)	129 (96.3%)	3 (15%)	65 (95.6%)	0 (0%)	64 (96.9%)
Sensitivity	88%		85%		100%	
Specificity	96.27%		95.59%		96.97%	
Positive predictive value	81.45%		85%		71.43%	
Negative predictive value	97.73%		95.59%		100%	

When the sensitivity and specificity of MTAP immunohistochemical staining loss were calculated as an alternative to the *CDKN2A* FISH in all cases, the sensitivity for the first observer was 88.00%, and the specificity was 95.52%. In comparison, the sensitivity for the second observer was 88.00%, and the specificity was 96.27%. Among the astrocytomas, the sensitivity of MTAP for the first observer was 90%, and the specificity was 95.59%, while the sensitivity for the second observer was 85%, and the specificity was 95.59%. In the oligodendrogliomas, the sensitivity of MTAP for the first observer was 80%, and the specificity was 95.45%, while the sensitivity for the second observer was 100%, and the specificity was 96.97%.

**Figure 2.**
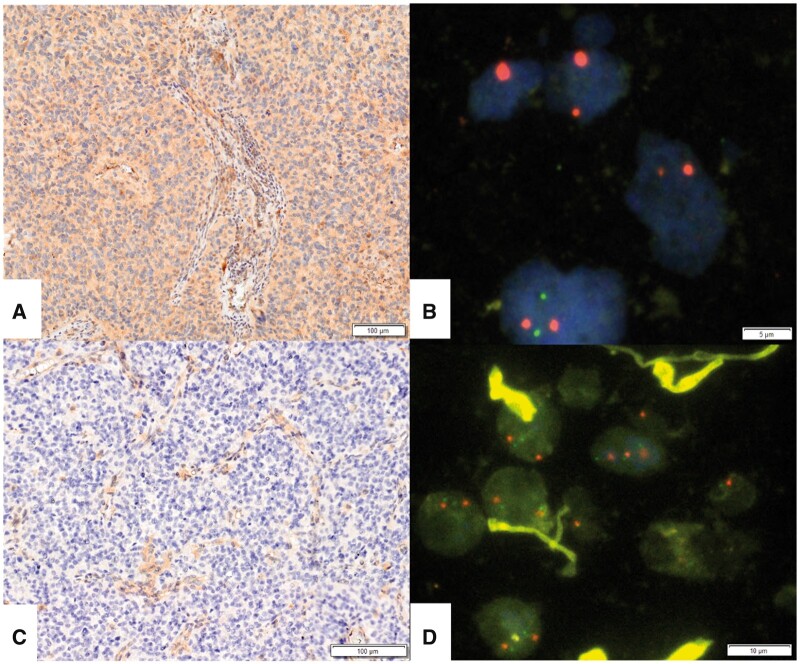
Discrepancy between *CDKN2A* FISH and MTAP IHC. First case; **(A)** retained MTAP expression but **(B)***CDKN2A* HD. Second case; **(C)** Loss of MTAP expression but **(D)***CDKN2A* normal. In FISH images, green signals represent *CDKN2A*; orange signals represent *CEN9*.

There were also cases showing discordance between *CDKN2A* status and MTAP expression. MTAP loss was observed in 22 of 25 tumors that have *CDKN2A* HD; but not detected in 3 of them (Fig. 2A, B). Meanwhile, MTAP expression loss was noted in 5 of 134 tumors without *CDKN2A* HD (Fig. 2C, D). When the relationship between *CDKN2A* HD or MTAP loss and patient age, gender, and tumor location was analyzed, no correlation was found.

## DISCUSSION

Adult-type IDH-mutant gliomas are divided into 2 major groups on the basis of 1p/19q status: IDH-mutant astrocytoma, and IDH-mutant and 1p/19q codeleted oligodendroglioma. According to the recent change of IDH-mutant astrocytoma grading in the 2021 WHO Classification of CNS tumors, tumors containing *CDKN2A/B* HD are accepted as grade 4 even if they do not contain MVP and/or necrosis (1). Therefore, assessment of *CDKN2A/B* HD status is mandatory for accurate grading of diffuse astrocytic tumors lacking necrosis and/or MVP. In oligodendrogliomas, *CDKN2A/B* HD, which is not seen in WHO CNS grade 2 tumors but can be seen in some grade 3 tumors, has been found to be related to short survival times independent of MVP and necrosis (8).

Overall, we detected *CDKN2A* HD in 25 (15.7%) of 159 tumors. This rate was similar to the “French national POLA network” cohort (13.9%, 127/911) reported by Appay et al (8). *CDKN2A* HD was detected in 20 (22.7%) of 88 astrocytoma cases. Although this rate was higher than the rates of the cohorts of Appay et al (11%, 47/428) and Shirahata et al (5) (14%, 31/224); it was lower than the cohort of Korshunov et al (26) (43%, 42/97). Due to the presence of *CDKN2A* HD, the CNS WHO grade of 10 (11.3%) IDH-mutant astrocytomas was upgraded to grade 4 (2 were previously grade II, 8 were previously grade III according to the WHO 2016 at the time of diagnosis).

In oligodendrogliomas, *CDKN2A* HD was detected in 7.04% (5/71) of tumors; all were WHO CNS grade 3 tumors. *CDKN2A* HD was not detected in any of the grade 2 tumors. In 2020, Appay et al (8) reported that *CDKN2A* HD was seen in 7% (33/483) of grade 3 oligodendrogliomas, similar to our result, and *CDKN2A* HD in oligodendroglial tumors was associated with shorter overall survival independent of necrosis and MVP. Therefore, based on these results, it might be useful to determine *CDKN2A* HD status in oligodendroglial tumors with difficulty in grading.

There are suggestions in the literature that MTAP immunohistochemical staining can be utilized as a surrogate biomarker for molecular methods in detecting *CDKN2A* HD (14–21). However, there are a limited number of studies conducted on diffuse gliomas (24, 25). In our study, the sensitivity of MTAP was 88% for both observers, and the specificity was 95.52%-96.27%, respectively. This finding is consistent with the 2 other studies in diffuse gliomas reported by Satomi et al (24) (88% sensitivity and 98% specificity), and Maragkou et al (25) (97% sensitivity and 100% specificity). Similarly, in a recent study with 23 PXA cases, the sensitivity of MTAP IHC was found to be 86.7% and the specificity 100% (18). In a study of 30 meningioma cases published by Sasaki et al (20), MTAP loss was found in all 5 cases in which *CDKN2A* HD was detected, and they reported sensitivity and specificity of MTAP as 100%. With these findings, we can conclude that MTAP IHC is a reliable surrogate biomarker that shows high sensitivity and specificity for detecting *CDKN2A* HD in diffuse gliomas as well as other CNS tumors.

However, some cases show a discrepancy between *CDKN2A* status and MTAP expression. In our study, loss of MTAP staining was observed in 22 of 25 cases that have *CDKN2A* HD; in 3 of them, MTAP loss was not detected. In 80%-90% of cases, deletion in chromosome 9p21 is associated with HD of *CDKN2A* and HD of *MTAP* gene, which is 165 kb telomeric to *CDKN2A* (22). However, depending on the extent of the deletion, sometimes *CDKN2A* HD may not be accompanied by *MTAP* HD. According to a study including 230 glioma cases, among 9p21 deleted cases 10% had *CDKN2A* HD without *MTAP* HD (23). In a study with pancreatic and periampullary cancers, in which *CDKN2A* HD is common (40%), it was reported that *CDKN2A* HD was seen in half of the cases without *MTAP* HD (16). Therefore, in some cases, there is no immunohistochemical loss of MTAP as *MTAP* HD does not accompany existing *CDKN2A* HD. Based on these findings, this situation might be valid for the 3 cases in our series that did not show MTAP loss despite *CDKN2A* HD. The conclusion to be drawn here is that retained MTAP expression does not always rule out *CDKN2A* HD.

In our series, MTAP expression loss was observed in 5 of 134 cases without *CDKN2A* HD. In the literature, rare cases (0.5%) of *MTAP* HD without *CDKN2A* HD have been reported (23). This situation might be explained either by *MTAP* HD without *CDKN2A* HD or another MTAP protein inactivation mechanism rather than HD (27). In conclusion, loss of MTAP staining does not always indicate the presence of *CDKN2A* HD.

Interobserver agreement of MTAP expression loss between 2 independent observers was calculated by Kappa coefficient analysis in our study; an almost perfect agreement of 0.938 was found. Similarly, Chapel et al (14) showed very good and good agreement, respectively, between 4 observers (Kappa coefficient 0.85) and 2 laboratories (Kappa coefficient 0.77) in MTAP staining in mesothelioma cases. Therefore, MTAP IHC emerges as a reliable surrogate biomarker with high interobserver agreement.

The 2 most used clones for MTAP IHC are “EPR6893” and “2G4.” In this study, clone EPR6893 was used, and immunohistochemical staining was able to be evaluated optimally in neoplastic cells and internal controls in most of the existing cases. Similarly, Lou et al (18) reported that they obtained effective staining with the “EPR6893” clone in PXA cases. However, Satomi et al reported that they could easily evaluate 61% of the cases with the “EPR6893” clone and 64% with the “2G4” clone in their diffuse glioma series. They also mentioned that the staining was stronger with the “2G4” clone even though they encountered similar difficulties for both clones when scoring the staining (24). The major problem in assessing the MTAP IHC is interpreting the absence of staining. Insufficient tissue fixation and technical issues during the staining procedure may lead absence of MTAP staining which can be interpreted as a false positive result. It is crucial to ensure that internal controls such as endothelial, inflammatory, and non-neoplastic glial cells are stained before evaluating MTAP loss. It will be wise to perform *CDKN2A* FISH in the suspicious cases.

**Figure 3.**
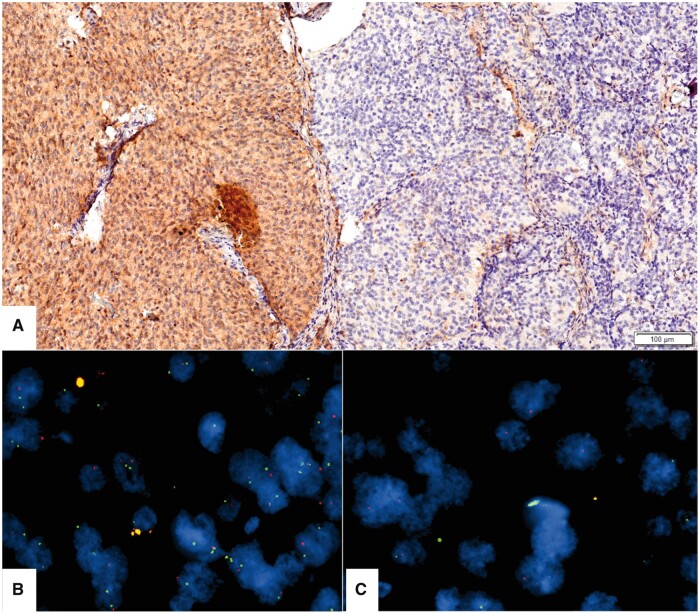
Demonstration of intratumoral heterogeneity. **(A)** There is a sharp demarcation between lost and retained expression of MTAP staining. **(B)***CDKN2A* HD is not seen in tumor cells with retained MTAP expression. **(C)** There is *CDKN2A* HD in tumor cells with MTAP loss. In the FISH images, green signals represent *CDKN2A*; orange signals represent *CEN9*.

To observe intratumoral heterogeneity in MTAP IHC and *CDKN2A* FISH, TMA blocks were prepared by taking 2 separate cores from different areas for each tumor. In the evaluation of MTAP IHC, intratumoral heterogeneity was observed in 4 cases, while MTAP immunohistochemical staining was homogeneous for both cores in the remaining cases. Three of 4 cases showed intermingling stained and non-stained cells. Only in one case, a sharp demarcation of loss of MTAP expression supported by *CDKN2A* FISH heterogeneity was observed. As illustrated in Figure 3, *CDKN2A* HD was absent in tumor areas where MTAP expression was preserved and *CDKN2A* HD was observed in tumor cells with MTAP loss. The remaining 158 cases did not show intratumoral heterogeneity in the *CDKN2A* FISH examination. In contrast to our results, Maragkou et al (25) reported that they did not observe any heterogeneity in MTAP staining in their glioma cohort. However, some studies using DNA methylation techniques have reported that intratumoral heterogeneity can be seen in diffuse gliomas (28, 29). Among the intratumoral heterogeneity for *CDKN2A/B* HD, Vega et al (28) mentioned in their study that they evaluated intratumoral heterogeneity with the help of DNA methylation profile in 39 cases of glioma and meningioma, and they reported that they detected intratumoral heterogeneity in *CDKN2A/B* HD in 1 of the 2 IDH-mutant astrocytomas included in the study, and this may lead to incorrect grading of IDH-mutant astrocytomas. So, the intratumoral heterogeneity seen in MTAP staining in some cases may reflect the heterogeneity seen in *CDKN2A* HD. Therefore, the possibility of intratumoral heterogeneity should be kept in mind when evaluating MTAP IHC, as well as the assessment of *CDKN2A* HD by FISH.

In addition to accompanying *CDKN2A* HD, there are also publications indicating that MTAP inactivation contributes to gliomagenesis through epigenetic mechanisms, especially in glioblastomas (30); thus, MTAP may be a target for treatment (30, 31). In the future, with the elucidation of the underlying mechanisms and the development of new targeted therapies, MTAP might be used as a marker that has the potential to be encountered with its therapeutic features as well as its diagnostic and prognostic features.

In summary, MTAP immunohistochemical staining seems to be a reliable surrogate biomarker that has high sensitivity and specificity for detecting *CDKN2A* HD and shows high interobserver agreement. It is essential to be aware of intratumoral heterogeneity while evaluating both *CDKN2A* HD and MTAP loss in order to make a correct assessment.

## ETHICAL APPROVAL

This study was conducted with appropriate ethics committee approval: Hacettepe University Non-interventional Research Ethics Committee; 2021/17-08.

## References

[1] WHO Classification of Tumours Editorial Board. Central Nervous System Tumours, 5th edition. Lyon (France): WHO Classification of Tumours Series; 2021

[2] Yan H , Parsons DW, Jin G, et al IDH1 and IDH2 mutations in gliomas. N Engl J Med 2009;360:765–73 19228619 10.1056/NEJMoa0808710 PMC2820383

[3] Reifenberger J , Reifenberger G, Liu L, et al Molecular genetic analysis of oligodendroglial tumors shows preferential allelic deletions on 19q and 1p. Am J Pathol 1994;145:1175–90 7977648 PMC1887413

[4] Reis GF , Pekmezci M, Hansen HM, et al CDKN2A loss is associated with shortened overall survival in lower-grade (World Health Organization Grades II-III) astrocytomas. J Neuropathol Exp Neurol 2015;74:442–52 25853694 10.1097/NEN.0000000000000188 PMC4397174

[5] Shirahata M , Ono T, Stichel D, et al Novel, improved grading system(s) for IDH-mutant astrocytic gliomas. Acta Neuropathol 2018;136:153–166 29687258 10.1007/s00401-018-1849-4

[6] Yang RR , Shi ZF, Zhang ZY, et al IDH mutant lower grade (WHO Grades II/III) astrocytomas can be stratified for risk by CDKN2A, CDK4 and PDGFRA copy number alterations. Brain Pathol 2020;30:541–553 31733156 10.1111/bpa.12801 PMC8018138

[7] Brat DJ , Aldape K, Colman H, et al cIMPACT-NOW update 5: Recommended grading criteria and terminologies for IDH-mutant astrocytomas. Acta Neuropathol 2020;139:603–608 31996992 10.1007/s00401-020-02127-9 PMC8443062

[8] Appay R , Dehais C, Maurage CA, et al CDKN2A homozygous deletion is a strong adverse prognosis factor in diffuse malignant IDH-mutant gliomas. Neuro Oncol 2019;21:1519–28 31832685 10.1093/neuonc/noz124 PMC7145561

[9] Sharpless NE. INK4a/ARF: A multifunctional tumor suppressor locus. Mutat Res 2005;576:22–38 15878778 10.1016/j.mrfmmm.2004.08.021

[10] Sherr CJ. The INK4a/ARF network in tumour suppression. Nat Rev Mol Cell Biol 2001;2:731–7 11584300 10.1038/35096061

[11] Rao LS , Miller DC, Newcomb EW. Correlative immunohistochemistry and molecular genetic study of the inactivation of the p16INK4A genes in astrocytomas. Diagn Mol Pathol 1997;6:115–22 9098651 10.1097/00019606-199704000-00008

[12] Burns KL , Ueki K, Jhung SL, et al Molecular genetic correlates of p16, cdk4, and pRb immunohistochemistry in glioblastomas. J Neuropathol Exp Neurol 1998;57:122–30 9600204 10.1097/00005072-199802000-00003

[13] Purkait S , Jha P, Sharma MC, et al CDKN2A deletion in pediatric versus adult glioblastomas and predictive value of p16 immunohistochemistry. Neuropathology 2013;33:405–12 23311918 10.1111/neup.12014

[14] Chapel DB , Schulte JJ, Berg K, et al MTAP immunohistochemistry is an accurate and reproducible surrogate for CDKN2A fluorescence in situ hybridization in diagnosis of malignant pleural mesothelioma. Mod Pathol 2020;33:245–254 31231127 10.1038/s41379-019-0310-0

[15] de Oliveira SF , Ganzinelli M, Chilà R, et al Characterization of MTAP gene expression in breast cancer patients and cell lines. PLoS One 2016;11:e0145647 26751376 10.1371/journal.pone.0145647 PMC4709099

[16] Hustinx SR , Hruban RH, Leoni LM, et al Homozygous deletion of the MTAP gene in invasive adenocarcinoma of the pancreas and in periampullary cancer: A potential new target for therapy. Cancer Biol Ther 2005;4:83–6 15662124 10.4161/cbt.4.1.1380

[17] Hustinx SR , Leoni LM, Yeo CJ, et al Concordant loss of MTAP and p16/CDKN2A expression in pancreatic intraepithelial neoplasia: Evidence of homozygous deletion in a noninvasive precursor lesion. Mod Pathol 2005;18:959–63 15832197 10.1038/modpathol.3800377

[18] Lou L , Li J, Qin M, et al Correlation of MTAP immunohistochemical deficiency with CDKN2A homozygous deletion and clinicopathological features in pleomorphic xanthoastrocytoma. Brain Tumor Pathol 2023;40:15–25 36550382 10.1007/s10014-022-00447-0

[19] Powell EL , Leoni LM, Canto MI, et al Concordant loss of MTAP and p16/CDKN2A expression in gastroesophageal carcinogenesis: Evidence of homozygous deletion in esophageal noninvasive precursor lesions and therapeutic implications. Am J Surg Pathol 2005;29:1497–504 16224217 10.1097/01.pas.0000170349.47680.e8

[20] Sasaki S , Takeda M, Hirose T, et al Correlation of MTAP immunohistochemistry with CDKN2A status assessed by fluorescence in situ hybridization and clinicopathological features in CNS WHO grade 2 and 3 meningiomas: A single center cohort study. J Neuropathol Exp Neurol 2022;81:117–126 34897475 10.1093/jnen/nlab127

[21] Su CY , Chang YC, Chan YC, et al MTAP is an independent prognosis marker and the concordant loss of MTAP and p16 expression predicts short survival in non-small cell lung cancer patients. Eur J Surg Oncol 2014;40:1143–50 24969958 10.1016/j.ejso.2014.04.017

[22] Zhang H , Chen ZH, Savarese TM. Codeletion of the genes for p16INK4, methylthioadenosine phosphorylase, interferon-alpha1, interferon-beta1, and other 9p21 markers in human malignant cell lines. Cancer Genet Cytogenet 1996;86:22–8 8616780 10.1016/0165-4608(95)00157-3

[23] Ceccarelli M , Barthel FP, Malta TM, et al; TCGA Research Network. Molecular profiling reveals biologically discrete subsets and pathways of progression in diffuse glioma. Cell 2016;164:550–63 26824661 10.1016/j.cell.2015.12.028 PMC4754110

[24] Satomi K , Ohno M, Matsushita Y, et al Utility of methylthioadenosine phosphorylase immunohistochemical deficiency as a surrogate for CDKN2A homozygous deletion in the assessment of adult-type infiltrating astrocytoma. Mod Pathol 2021;34:688–700 33077924 10.1038/s41379-020-00701-w

[25] Maragkou T , Reinhard S, Jungo P, et al Evaluation of MTAP and p16 immunohistochemical deficiency as surrogate marker for CDKN2A/B homozygous deletion in gliomas. Pathology 2023;55:466–477 37032198 10.1016/j.pathol.2023.01.005

[26] Korshunov A , Casalini B, Chavez L, et al Integrated molecular characterization of IDH-mutant glioblastomas. Neuropathol Appl Neurobiol 2019;45:108–118. 10.1111/nan.12523 30326163

[27] Schmid M , Malicki D, Nobori T, et al Homozygous deletions of methylthioadenosine phosphorylase (MTAP) are more frequent than p16INK4A (CDKN2) homozygous deletions in primary non-small cell lung cancers (NSCLC). Oncogene 1998;17:2669–75 9840931 10.1038/sj.onc.1202205

[28] Ferreyra Vega S , Wenger A, Kling T, et al Spatial heterogeneity in DNA methylation and chromosomal alterations in diffuse gliomas and meningiomas. Mod Pathol 2022;35:1551–1561 35701666 10.1038/s41379-022-01113-8 PMC9596370

[29] Verburg N , Barthel FP, Anderson KJ, et al Spatial concordance of DNA methylation classification in diffuse glioma. Neuro Oncol 2021;23:2054–2065 34049406 10.1093/neuonc/noab134 PMC8643482

[30] Hansen LJ , Sun R, Yang R, et al MTAP loss promotes stemness in glioblastoma and confers unique susceptibility to purine starvation. Cancer Res 2019;79:3383–3394 31040154 10.1158/0008-5472.CAN-18-1010 PMC6810595

[31] Kindler HL , Burris HA III, Sandler AB, Oliff IA. A phase II multicenter study of L-alanosine, a potent inhibitor of adenine biosynthesis, in patients with MTAP-deficient cancer. Invest New Drugs 2009;27:75–81 18618081 10.1007/s10637-008-9160-1

